# How to Improve Interactions between Police and the Mentally Ill

**DOI:** 10.3389/fpsyt.2014.00186

**Published:** 2015-01-14

**Authors:** Yasmeen I. Krameddine, Peter H. Silverstone

**Affiliations:** ^1^Department of Psychiatry, University of Alberta, Edmonton, AB, Canada

**Keywords:** police, training, mental health, effective, program, research, review

## Abstract

There have been repeated instances of police forces having violent, sometimes fatal, interactions with individuals with mental illness. Police forces are frequently first responders to those with mental illness. Despite this, training police in how to best interact with individuals who have a mental illness has been poorly studied. The present article reviews the literature examining mental illness training programs delivered to law-enforcement officers. Some of the key findings are the benefits of training utilizing realistic “hands-on” scenarios, which focus primarily on verbal and non-verbal communication, increasing empathy, and de-escalation strategies. Current issues in training police officers are firstly the tendency for organizations to provide training without proper outcome measures of effectiveness, secondly the focus of training is on changing attitudes although there is little evidence to demonstrate this relates to behavioral change, and thirdly the belief that a mental health training program given on a single occasion is sufficient to improve interactions over the longer-term. Future police training needs to address these issues.

## Importance of Police Research

Police and other law-enforcement officers are frequently the first-line responders to those suffering from a psychiatric crisis. Unfortunately, negative interactions between individuals with mental illness and law enforcement are widely reported and frequently tragic. Mental health training is essential to reduce the number of undesirable outcomes between police and law-enforcement individuals and those suffering from mental illness, with research finding that a lack of training leads to an escalation in violence (1), and increased rates of injury and death (2–5). This offers the potential that with appropriate training of police officers, particularly focusing on better communication and the ability to more easily de-escalate emotions during these interactions that this will reduce the frequency of these negative interactions (6, 7).

Training police on how best to interact with individuals who may have a mental illness is not new. A recent study looking at Canadian law-enforcement organizations found that entry-level training on mental illness occurs widely and provides a strong groundwork for positive interactions, as well as noting significant increases in crisis intervention training in the last decade in many countries, including Canada, the United States, the United Kingdom, and Australia (8). Nonetheless, although training has increased, there continues to be a number of issues that remain. The present review focuses on recommendations for change, and includes recent suggestions for both police training and police organizations (8, 9). Taking all of these into consideration, the current review proposes a focus on specific aspects of training that must be enhanced to improve outcomes, and how this research should best be carried out in collaboration with police forces.

## Mental Health Training Program

We have previously developed a comprehensive 1-day mental health training program, created at the University of Alberta in partnership with the Edmonton Police Service (EPS) (10). In this program, police officers interacted with professional actors in a “hands-on” training program comprising six realistic mental health scenarios. Both direct and indirect measurements of changes in attitudes and behaviors were collected before and after training, allowing a detailed analysis of the impact of training (11).

The main training objective of the program was a focus on maintaining officer engagement throughout the training day. This was done by concentrating on improving officer communication (both verbal and non-verbal), de-escalation techniques, officer empathy, and symptom recognition in a unique way. One key aspect of this trianing program was the feedback given to each officer after every scenario. Each of the scenarios was designed to be highly emotionally arousing, and it was intended that by the end of six scenarios, in which the key messages were repeatedly reinforced, there would be stronger retention of what was taught. Feedback was given by four separate individuals, a senior facilitating officer (focusing on officer performance and safety), a mental health professional [consisting of a member from the EPS police and crisis team (PACT) who gave recommendations and addressed concerns and questions], and the professional actors (one acting and another one watching) who were trained to provide feedback regarding police behavior.

KEY CONCEPT 1. Engage officers Training must be both emotionally and intellectually engaging. This can be done by using a role play-based, hands-on approach with scenarios using professional actors with a focus on communication, de-escalation, and empathy.

Actor feedback focused on positive behaviors as well as behaviors that could be improved upon such as non-verbal communication skills (body language, facial language, and active listening) and verbal communication skills (tone of voice, word choice, portrayed empathy, rapport, and de-escalation techniques). This feedback provides a unique link for each officer regarding how their behavior affected those they interact with, and increased their behavioral self-awareness.

The outcome of this training was very positive, and after training over 650 officers significant behavioral improvements were still present 6 months later (11). The behavioral changes were noted primarily by the supervising officers, who found that police patrol officers were significantly better in communication, empathy, and de-escalation techniques following training. Additionally, measurements of the average number of mental health calls increased by over 40%, emphasizing an enhanced ability of officers to recognize a mental health issue. Furthermore, police officers also spent nearly 20% less time on each mental health call, supporting an improvement in communication, empathy, de-escalation, and knowledge of appropriate solutions, which translated into a more effective interaction with improved efficiency of these. Supporting the positive outcome of the training, police officers self-reported a 23% increase in confidence when interacting with individuals in psychiatric distress.

This overall increase in police efficiency over a 6-month period led to over $80,000 in cost savings. Lastly, there was a more than 40% decrease in any use of force following training, although it should be noted that there were other specific initiatives to try and address this issue that were co-occurring and may also have led to this decrease in use of force, and it was therefore unlikely that the training program was the sole reason for this decrease in the use of force.

Interestingly, no changes in police attitudes were found 6 months after training, although this was expected since training focused on behavior and not attitudes. This is of importance, as it demonstrates that attempting to change attitudes may not be the most effective approach, while methods to change behaviors alone (without focusing on attitudes) can be very effective. Taken together, these outcomes emphasize the positive impact of this focused training program.

## Current Limitations of Training Programs

### Need to focus training programs on outcomes

Current police training focuses on symptom recognition, crisis management techniques, and increased communication between law enforcement and mental health services (12). Findings have shown improvements in confidence levels of officers (11, 13, 14), increases in positive attitudes/decreases in stigma toward mental illness (13, 15, 16), and more optimal changes in behavior (1, 11, 15, 17, 18). Despite these positive findings, there are other initiatives that have not found any positive changes following training (15, 19–21). As previously noted, while police training on mental illness is common, with a variety of training programs worldwide (22), only a very few of these programs have been properly evaluated to determine that they lead to meaningful behavioral changes (23). For example, although several programs describe changes in attitudes and thoughts of police and law-enforcement individuals toward those with mental illness, on its own this is not sufficient information to demonstrate a positive outcome. It has been frequently noted that if any training program lacks an appropriate research design, the effectiveness of such a program cannot be tested (7). Thus, these programs, usually associated with high costs, fail to establish a cost–benefit relationship, which does not allow a determination of if they are successful or not.

KEY CONCEPT 2. Outcomes The effectiveness of each training program must be analyzed. This can be done using proper outcome measures, some of which are listed below.

To ensure some consistency between police forces, both in the items measured and recommendations made, we have a total of 10 suggestions that we believe should be carried out consistently when police training is given in this area. Training efficacy can then be reliably and reproducibly measured, both within a specific police force and between them. These suggestions include outcome measurements used in our recent study:

1. The number of mental health calls that police attend.
2. The time required during each mental health call.
3. The number of use of force occurrences in mental health calls.
4. Supervisor ratings of officers for empathic communication (from 0 to 10).
  As well as recommendations from other reviews (8):
5. Satisfaction measures of mentally ill individuals that interacted with a police officer (although it should be noted that this can be difficult to measure consistently, however).
6. Satisfaction measures of community and mental health services that interacted with a police officer.
7. Number of arrests compared to the total number of mental health interactions.
8. Number of injuries during a police interaction with those who may have a mental illness.
  Other recommendations for measurement:
9. Overall number of complaints.
10. Officer self-reported measurements of their behavior (since findings have shown that self-reported measurements are 11% more accurate then those rated by observers) (24).

By incorporating this data, along with detail regarding what each training program consisted of, more information can be collected to determine overall efficiency of programs. This would allow police forces and other law-enforcement agencies to determine if their current training was appropriate and having the desired impact.

### Need to focus training programs on behavioral change not attitudinal change

Another major issue in current police training revolves around the assumption that if attitudes toward mental illness can be made more positive then behaviors will change accordingly. Because of this assumption, current training programs focus on changing attitudes through educational means even though their main goal is to change behaviors. In this regard, we must understand the challenge it takes in changing attitudes. Once attitudes, stereotypes, or biases are established, they are extremely difficult to modify (25). As well, if attitudes are strong, behaviors are increasingly more difficult to change. If the end goal is to improve an officer’s behavior toward individuals with mental illness, a more efficient way is to focus on changing behavior, assuming attitudes will change accordingly. This theory is termed cognitive dissonance or self-justification. When attitudes and behaviors are inconsistent with each other, individuals have beliefs that attitudes and behaviors should be related and thus aim to diminish tension by shifting their attitudes to match their behaviors (26). Attitudes only change if officers are unable to justify externally, why they acted in a certain way. For example, if a sergeant was watching, officers justify their actions by telling themselves they acted this way because the sergeant was watching. However, if behaviors are implemented without external justification, then there will be an internal attitude shift linking behaviors to attitudes. For example, officers will believe that the reason they acted this way was because they like acting this way, leading to an attitude change.

KEY CONCEPT 3. Behaviors Need to focus on changing behaviors and not just attitudes toward mental illness. The relationship between attitudes and behaviors is complex and one does not necessarily lead to changes in the other.

Interestingly, although it is difficult to accomplish, some research does show improvements in police attitudes and stigma toward mentally ill individuals and positive behavioral changes after training. However, even if attitudes and behaviors do change post training, there is evidence showing that attitudes do not always predict behaviors and vice versa.

There are four factors that strengthen or weaken the link between attitudes and behavior:

1. Specificity: specific attitudes must be compared to specific behaviors, and general attitudes must be compared to general behaviors. If there is a mismatch then actual attitudes may not be determined (27, 28). For example, general attitude toward mental illness will not predict behaviors toward depressed individuals.
2. Individual differences: some individuals are able to change their behavior according to the situation (high self monitors), while others act the same in all situations (low self monitors). Low self-monitors act according to their attitudes. (29). It has been found that if individual confidence is increased, people can become high self-monitors (30).
3. Attitude strength: the stronger the attitude toward something makes the attitude readily accessible and a greater predictor of behavior (31). Attitudes can be strengthened through direct or personal experience. As well, the stronger the attitude, the more difficult it is to change the attitude.
4. Conformity and obedience: if individuals are forced to behave in accordance with group norms or commanding officer beliefs, it is less likely that behaviors match private attitudes because they may be complying to avoid punishment or gain reward (32).

Two models that describe this relationship further using psychological processes are the attitude-to-behavior process model and the theory of planned behavior.

The attitude-to-behavior process model explains spontaneous behavior in response to an unexpected situation. It states that more accessible attitudes have instantaneous effects on behavior (33). Explaining behavior in terms of the specific situations tends to be overlooked (32) and instead behaviors are explained in terms of personality and attitudes, thus attitude–behavioral relationships are made even if they may not exist (32). Even so, we must not ignore the influence that the specific situation has on behavior.

The theory of planned behavior is a more complex theory that links attitudes and behaviors. This model proposed by Ajzen (27, 34) describes the main determinant of behavior to be behavioral intention, which is composed of three main predictors:

1. strength of attitudes toward the behavior,
2. subjective norms (i.e., an individual’s perception of social pressure to perform the targeted behavior), and
3. perceived behavioral control (i.e., how much control the person believes they have over the behavior, and how confident a person feels about being able to perform the behavior).

However, even if these three factors are strengthened to positively influence behavior, behavioral intention is only found to predict variance in behaviors 19–36% of the time (24, 35–37). Although there are limitations to this theory, the theory of planned behavior is one of the most widely used and predictive influential theories, and has been used successfully to describe and predict behaviors (37). Thus, even if police officers have better or worse attitudes toward those with mental illness, there is no guarantee that they will behave more positively (or negatively). This is consistent with the findings from our studies, which have shown that behavioral change in this group is independent of changes in attitude (10, 11), where initial attitudes officers held toward mental illness were consistent with those of control populations (38–40). This is supported by suggestions that training should primarily focus on changing behaviors of officers instead of attitudes (41). Nonetheless, it remains important to address attitudes about mental illness in a positive manner to avoid negative stereotypes toward those suffering from mental illness. To increase the probability that officers have positive and empathetic views of mental illness, attitudes must be addressed in the early stages of training. By priming officers toward a greater understanding of mental illness, negative attitudes may be prevented from forming.

In this program, we have outlined, police officers received multiple different feedbacks. While the impact of the feedback is clear, it is not possible to determine what effect each individual type of feedback is having or if one type is the most important of these. The goal remains to change behavior, and adopting training approaches that target changing behavior can therefore produce better results. Further research is needed to determine the impact of critical and constructive criticisms of police officers on their behavior, and it may be of help to more specifically refocus the feedback from actors to be more in line with the behavioral change desired in police officers. Scripting such feedback more closely could also potentially improve outcomes. All of these issues warrant further research, and in this regard, it should be acknowledged that any training program requires continual evaluation/research to determine what elements are most important, and what can be best improved.

### A training program needs to be repeated

A third issue with current training programs is the lack of repeated, or refresher, training. While police forces recognize the need for regular and repeated training on a range of areas, this does not seem to apply to the issue of interactions with the mentally ill, where single training activities are the norm. This is despite compelling research regarding memory retention, which suggests a challenge for even the most intelligent students to remember material over time. As an example, medical students forget 25–35% of material in the first year, and more than 50% by the second (42). Another review suggests that memory is imperfect, and that skills and knowledge decay by 6 months to 1 year post training, with skills deteriorating faster than knowledge (43).

KEY CONCEPT 4. Repeated Training must be repeated to optimize the effects of both skills acquired and memory retention. This can be done with refresher training every 1–3 years.

Other evidence regarding health related skills and knowledge retention suggest that refresher training should occur at least every 3 years (44–47). Additional support for the need for police organizations to implement repeated training in this area is research showing that police retention of knowledge decreases over time (48). For these reasons, training on mental health awareness needs to be repeated regularly, with current evidence suggesting training must occur every 3 years for all individuals involved in interactions with those who may have a mental illness.

## Future Directions

Current recommendations for police training continue to emphasize the importance of training law-enforcement officers to interact more appropriately with individuals suffering from mental illness. The future direction of training for these individuals needs to address the specific factors identified in this review. First, these are the need to accurately measure the outcomes from training. Without this, it is impossible to determine if any training programs are successful. Considering the large sums of money and time it takes to carry out a training program, outcome measures are increasingly important for all police training programs, and this should also apply to those involving training for interacting with mentally ill individuals. Second, there is a need for training programs to focus on changing behaviors and not simply attitudes, since attitudes, and behaviors may not be strongly correlated. Evidence to date suggests that this can be achieved by focusing on communication, empathy, and de-escalation by engaging officers through scenario based, hands-on training. Third, it is essential to continuously train officers throughout their careers, and to work to maintain these skills and specific knowledge, preferably by having a training program every 3 years. By continuing the opportunity for officers to increase their mental health awareness, improvements in the relationship between police and mentally ill individuals will continue to progress over time. Officers will then be better equipped to know what to look for, to ask the right questions, and to behave appropriately toward individuals with these conditions, thus increasing the number of positive interactions between these two groups.

## Conclusion

Appropriate police training is becoming recognized as a critical component in improving interactions between officers and those with mental illness. To minimize the number of tragic outcomes, it is important to continue to improve this training. Evidence to date supports the use of a scenario-based training program using testing methods supported by research-based outcomes. We suggest that this training program, and others like it, need to become widely used in the training of police officers to help them interact with those individuals who have a mental illness.

## Conflict of Interest Statement

The authors declare that the research was conducted in the absence of any commercial or financial relationships that could be construed as a potential conflict of interest.
